# Computational Modeling and Neuroimaging Techniques for Targeting during Deep Brain Stimulation

**DOI:** 10.3389/fnana.2016.00071

**Published:** 2016-06-30

**Authors:** Jennifer A. Sweet, Jonathan Pace, Fady Girgis, Jonathan P. Miller

**Affiliations:** Department of Neurosurgery, University Hospitals Case Medical Center, Case Western Reserve UniversityCleveland, OH, USA

**Keywords:** deep brain stimulation, computational modeling, neuroimaging, tractography, targeting

## Abstract

Accurate surgical localization of the varied targets for deep brain stimulation (DBS) is a process undergoing constant evolution, with increasingly sophisticated techniques to allow for highly precise targeting. However, despite the fastidious placement of electrodes into specific structures within the brain, there is increasing evidence to suggest that the clinical effects of DBS are likely due to the activation of widespread neuronal networks directly and indirectly influenced by the stimulation of a given target. Selective activation of these complex and inter-connected pathways may further improve the outcomes of currently treated diseases by targeting specific fiber tracts responsible for a particular symptom in a patient-specific manner. Moreover, the delivery of such focused stimulation may aid in the discovery of new targets for electrical stimulation to treat additional neurological, psychiatric, and even cognitive disorders. As such, advancements in surgical targeting, computational modeling, engineering designs, and neuroimaging techniques play a critical role in this process. This article reviews the progress of these applications, discussing the importance of target localization for DBS, and the role of computational modeling and novel neuroimaging in improving our understanding of the pathophysiology of diseases, and thus paving the way for improved selective target localization using DBS.

## Introduction

Deep brain stimulation (DBS) has been used to treat neurological disorders for several decades, but we still have little understanding of the mechanism by which it reverses pathological brain activity. This is in part due to the inherent difficulty in visualizing aberrant neural networks, thus challenging our ability to modulate such pathways via electrical stimulation. However, innovative developments in stereotaxy, computational modeling, engineering, and neuroimaging techniques have made it possible to identify complex connections of circuitry within the brain, improving our understanding of the pathophysiology of various diseases and facilitating our ability to influence these aberrant processes to produce therapeutic results. With continued advancements in engineering and neuroimaging, which enhance our targeting and delivery of stimulation, patients currently treated with DBS may experience improved, patient-specific outcomes, and those with other neurologic and psychiatric disorders may soon become candidates for surgical intervention using DBS.

## Mechanism of Action of DBS

DBS involves the surgical implantation of electrodes into deep structures of the brain to modulate brain circuitry in an effort to restore normal physiological function. DBS has been used effectively for the treatment of movement disorders, including Parkinson’s disease (PD), Essential tremor (ET), and dystonia, as well as for psychiatric disorders such as obsessive compulsive disorder (OCD). It is currently under investigation for the treatment of additional psychiatric disorders, cognitive dysfunction, epilepsy, and other potentially neurologically-mediated diseases. However, our lack of understanding of the exact mechanism of action of DBS remains a limiting factor in our ability to successfully treat such disease entities.

Given that the clinical effects of DBS produce outcomes comparable to those seen with lesioning techniques, the postulated mechanism of action of DBS was initially thought to be due to widespread neuronal inhibition (Kern and Kumar, 2007; Johnson et al., 2008). Such inhibitory effects were thought to occur from a depolarization blockade or a jamming of neural networks (Grafton et al., 2006; Kern and Kumar, 2007; Hamel et al., 2003). Interestingly, despite various studies demonstrating decreased activity of the targeted cells with high-frequency DBS, the output from these cells is not necessarily lessened (Johnson et al., 2008). In fact, studies in which the activity of cells receiving input from a stimulated target were recorded suggest that the stimulated cells may release more neurotransmitters with electrical stimulation (Hashimoto et al., 2003). Despite such seemingly contradictory evidence, it is now appreciated that the delivery of stimulation is vastly more complex, resulting in the activation of some elements and the inhibition of others, each of which results in a multitude of downstream effects (Lozano et al., 2002; Kringelbach et al., 2007; Kahan et al., 2012).

In addition, DBS may exert its influence via the correction of aberrant neuronal activity. For example, in the setting of DBS for the treatment of PD, the loss of dopamine, which is known to be largely responsible for the pathophysiology of the disease, results in changes in the underlying activity of cells within the basal ganglia (Galvan et al., 2015). Analyses of the electrical activity of neurons using local field potentials, electroencephalography, and electrocorticography have demonstrated alterations of inter-spike intervals, specific firing patterns, and oscillatory activities of neuronal cells in PD (Galvan et al., 2015). It is now believed that DBS acts to disrupt the abnormal activity of these cell populations, for instance, interfering with pathologically synchronous cell groups, thus further contributing to the clinical efficacy of stimulation (Lozano et al., 2002; Kahan et al., 2012).

Finally, our notion of the target of stimulation has also changed over time. While it was once assumed that DBS affected only the regional gray matter structures in which the electrodes were placed, it is currently recognized that the effects of the electrical current are more far-reaching, modulating nearby white matter tracts as well as local and distant cortical and subcortical structures (Miocinovic et al., 2006; Kahan et al., 2012; Sweet et al., 2014b). Again looking at the model of DBS for PD, investigations have demonstrated similar motor outcomes with stimulation of either the globus pallidus interna (GPi) or the subthalamic nucleus (STN; Burchiel et al., 1999; Follett et al., 2010). Neuroimaging studies performed on patients following DBS of the STN demonstrate that the therapeutic contact is frequently positioned at the dorsolateral border of the STN and overlaps with white matter tracts from the zona incerta and fields of Forel H2, which carry fibers from the GPi (Miocinovic et al., 2006; Hamel et al., 2003). Thus, stimulation of adjacent white matter tracts, potentially affecting larger neuronal networks, may in part explain the similar effects of DBS with STN and GPi stimulation.

This concept of the activation of diffuse fiber pathways has been demonstrated in numerous imaging studies, in which widespread alterations in blood flow, glucose metabolism, and blood oxygenation level dependence (BOLD) across the brain as a result of DBS are shown, again suggesting its likely influence on the connectivity of networks (Mayberg, 1997; Grafton et al., 2006; Laxton et al., 2010; Kahan et al., 2012; Figee et al., 2013; Van Hartevelt et al., 2015). In addition, computational models replicating the effects of DBS on cell body firing patterns and subsequent downstream axonal activation reveal that stimulation of the STN influences the firing of cells within the GPi, thus altering the output of pallidothalamic projection fibers, which in turn affect thalamic processing. Once again, this reinforces the idea that DBS modulates complex neuronal systems (McIntyre et al., 2004; Rubin and Terman, 2004; Miocinovic et al., 2006; Maks et al., 2009; Hahn and McIntyre, 2010).

However, despite our incomplete understanding of the mechanism of action of DBS, there is substantial evidence to show its clinical efficacy for the treatment of movement disorders. In 1998, Limousin et al. (1998) assessed 24 patients with advanced motor symptoms of PD, who underwent bilateral STN DBS for 1 year. The patients had a 60% improvement in their motor scores of dopaminergic medications, and their daily activities also significantly improved. Moreover, the mean dose of medication was reduced by half and there was no change in their cognitive outcomes. Other studies have demonstrated similar results (Burchiel et al., 1999; Volkmann et al., 2001; Walter and Vitek, 2004), and a long-term study of 42 PD patients with STN DBS assessed for 5 years showed sustained efficacy (Krack et al., 2003). In a randomized controlled trial in 2006, Deuschl et al. (2006) found a 25% improvement in quality of life and 41% improvement in motor symptoms in patients with PD, which was superior to the best medical management. Similarly, in a randomized open-label trial, DBS of the STN plus medical treatment had better outcomes medical treatment alone for PD (Williams et al., 2010).

These impressive outcomes are not unique to the treatment of PD, as such improvements in motor symptoms have also been shown in patients with ET and dystonia. In another randomized controlled trial, patients with severe tremor due to PD and ET were implanted with electrodes into the ventrointermediate (VIM) nucleus of the thalamus. The authors found almost complete cessation in tremor in 90% of patients (Schuurman et al., 2000). Moreover, in a follow-up study of the ET patients, up to 7 years after DBS, a significant reduction in tremor score was maintained (Blomstedt et al., 2007; Kocabicak et al., 2015). Similarly, prospective randomized controlled trials assessing GPi DBS for primary dystonias also reveal significant benefits in motor symptoms and quality of life measures (Vidailhet et al., 2005; Kupsch et al., 2006; Panov et al., 2013), with sustained and even improved effects many years following surgical intervention (Vidailhet et al., 2007; Volkmann et al., 2012; Panov et al., 2013).

While there are fewer long-term studies investigating the effects of DBS for psychiatric disorders, the results are still promising. Several small studies evaluating DBS of the anterior limb of the internal capsule have been done for the treatment of OCD, based on data from capsulotomy lesions, demonstrating notable efficacy in treatment-refractory patients (Nuttin et al., 1999, 2003; Greenberg et al., 2006). Other targets have also been studied for OCD including the nucleus accumbens and the STN (Sturm et al., 2003; Mallet et al., 2008; Huff et al., 2010; Hamani et al., 2014; Kocabicak et al., 2015). Additionally, DBS for the treatment of psychiatric disorders, such as major depression (MD), have also been assessed. Targets for these various trials include the subgenual cingulate cortex, the nucleus accumbens, and the anterior limb of the internal capsule (Mayberg et al., 2005; Lozano et al., 2008; Malone et al., 2009; Kennedy et al., 2011; Bewernick et al., 2012; Holtzheimer et al., 2012). The fact that each of these studies, targeting different structures within the brain, resulted in comparable outcomes, again suggests that a complex and inter-related network is influenced by electrode stimulation.

However, while the smaller studies yielded encouraging findings, these results could not be replicated in larger, multi-center trials (Dougherty et al., 2015). This may in part be due to the heterogeneous patient population, high rates of concomitant psychiatric comorbidities among patients, or an overall still poor understanding of the involved neural circuitry (Coenen et al., 2011a,b). Moreover, given the vulnerability of patients with psychiatric disease and the inconsistent outcomes shown with surgical interventions, ethical considerations must be taken into account prior to proceeding to surgery for such disease states. The potentially devastating consequences of poor clinical judgment is evidenced by historical events that transpired during the psychosurgery era. Led largely by Walter Freeman in the 1940s and 1950s, the rampant and unregulated practice of the frontal lobotomy, adapted from the frontal leucotomy procedure that was initially described in Europe by Egas Moniz, was performed indiscriminately in patients of all ages with diverse symptomatology across the United States. These surgeries resulted in unacceptably high complication rates with minimal evidence to support their efficacy (Robinson et al., 2013). This underscores the importance of careful patient selection and the need for stringent pre-surgical criteria.

Therefore, although the exact mechanism of DBS remains unclear, our understanding of the complex interplay of systems involved continues to grow. In addition, there is a great deal of evidence demonstrating the benefits of DBS in appropriately selected patients. However, if we are to further optimize observable outcomes by delivering patient-specific stimulation or treating novel neurological and psychiatric diseases in a successful and consistent manner, then utilization of sophisticated and innovative targeting, engineering, and imaging techniques is required. Advancements in computational modeling and neuroimaging that can better demonstrate neuronal activity within the context of widespread connection networks has largely contributed to our progress thus far. Such strategies and technologies are discussed in the duration of this review.

### Surgical Targeting

#### History of Surgical Targeting

As noted in the preceding section, the mechanism by which DBS exerts its clinical effects is incompletely understood. However, we do know that both the therapeutic benefits as well as many of the adverse symptoms resulting from DBS depend largely on the location of the electrode contacts within the brain. Thus surgical targeting is an essential factor contributing to the success of a DBS procedure.

**Figure 1:**
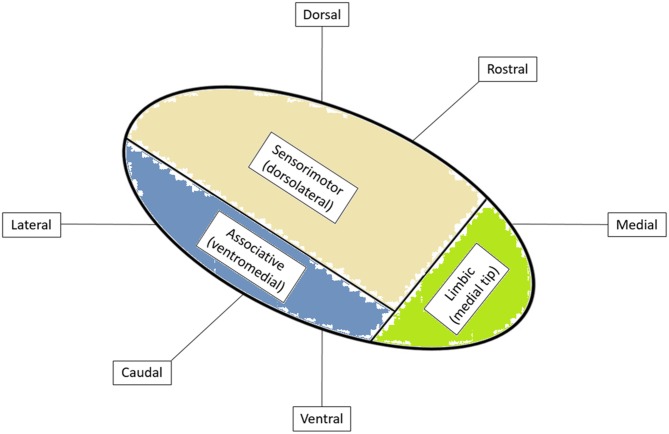
**Functional organization of subthalamic nucleus (STN) with cortical inputs into specific regions of the STN.** Adapted from Tewari et al. (2016).

Stereotaxis is the process by which deep regions of the brain may be surgically targeted via a minimally invasive approach using a three-dimensional coordinate system for spatial localization in reference to a targeting image (Schiefer et al., 2011). The role of stereotaxy for the treatment of neurological disorders dates to the 1940s when frame-based localization was used to aid in the precise targeting of intracranial structures (Leksell, 1949). The necessity for such precise and accurate localization is easily understood when considering the size and location of commonly targeted structures. For instance, the STN measures approximately 6 mm by 4 mm by 5 mm in size and is obliquely oriented in all three planes (Aboshch et al., 2010; Sweet et al., 2014a). In addition, the STN has been postulated to be somatotopically organized in a tripartite manner with respect to functional territories, such that the desired location of the DBS electrode is in the dorsolateral aspect of the nucleus, believed to correspond to the motor region of the STN (Figure 1; Joel and Weiner, 1997; Lambert et al., 2012; Tewari et al., 2016).

Historically, surgical targeting was done via a ventriculogram, to visualize the location of the anterior and posterior commissures which were used as reference points, and a standard stereotactic atlas, allowing for the determination of the desired target relative to its distance from these points (Walter and Vitek, 2004). The advent of computed tomography (CT) and magnetic resonance imaging (MRI), later replaced the use of the ventriculogram for indirect targeting. Yet, due to the potential error intrinsic to the process of indirect targeting, the practice of intraoperative microelectrode recording was developed. Microelectrode recording uses electrophysiology and somatosensory mapping to further confirm the precise location of the electrode within the brain (Walter and Vitek, 2004).

Further improvements in MRI technology and software targeting platforms has led to the practice of “direct” targeting, either alone or in combination with indirect targeting, to facilitate DBS electrode placement. This involves directly visualizing the target, such as the STN, using high-resolution MR images, rather than relying solely on the position of the target relative to the anterior and posterior commissures. Additional developments in neuroimaging, computational modeling, novel stimulation parameters, and improved electrode designs, are all drastically changing the way in which surgical targeting is performed. These developments aim to improve patient outcomes and aid in the understanding of both DBS and the pathophysiology of diseases.

#### High-Resolution MRI and Software Targeting Platforms

Improvements in neuroimaging modalities have revolutionized the process of DBS lead implantation. Advancements in MRI technology and the utilization of 3 tesla (T) MRI scanners rather than 1.5T MRI machines have made it possible to visualize deep structures of the brain to allow direct targeting of the structure with less reliance on indirect targeting based on standard landmarks (Rezai et al., 2006; Cho et al., 2010). Such visualization is even more impressive with 7-T MR systems, even allowing for detection of the VIM for ET (Aboshch et al., 2010; Cho et al., 2010).

In 2007, Guo et al. (2007) looked at six methods of targeting, including a T2-weighted MRI-based approach, a brain atlas, T1 and T2 maps, an electrophysiological database, a collection of final surgical targets from previous patients, and the combination of all of these functional and anatomical data. These were compared against the “gold standard,” expert localization with intraoperative electrophysiologic confirmation. The displacement between the planned targets and the actual surgical targets compared to the gold standard ranged from 1.7 to 3 mm, with the technique combining anatomical and functional data being the most accurate (Guo et al., 2007). Additional advances enabling MRI-guided stereotaxy using a clinical workstation has been shown in animal models to be accurate to within 0.3–0.5 mm (Bjarkam et al., 2009).

Integration of imaging techniques with computer software platforms can further facilitate identification of brain structures and aid in targeting. Many such systems allow for the fusion of multiple imaging sequences while also superimposing standard brain atlases (D’haese et al., 2005; Castro et al., 2006; Duay et al., 2008). In addition, fusing different neuroimaging methodologies such as CT and MRI have also been found to ameliorate target localization (Chen et al., 2011).

Such targeting platforms have also been integrated into both frame-based and frameless stereotaxy systems. Traditionally, the placement of stereotactic head frames followed by a volumetric head CT prior to DBS surgery has been used for determination of stereotactic coordinates within the frame space, and this has been shown to be a highly accurate method of stereotaxy (Acar et al., 2007; Balachandran et al., 2009). Yet, frame placement can be uncomfortable for the patient, and requires repeated imaging once the frame is placed prior to surgery, therefore potentially adding to the length of surgery. As such, novel technologies have been developed in an effort to alleviate these concerns, allowing for the accurate targeting of deep structures without a frame. “Frameless” skull-mounted stereotactic platforms have been evaluated and found to have comparable accuracy with frame-based systems, although the use of these systems require operating through a narrow working field that can make neurophysiological recordings, if performed, more challenging (Fukaya et al., 2010).

### Computational Modeling Techniques

#### Whole-Brain Activity Models

Computational modeling techniques have also contributed both to our understanding of and our progress in DBS. These techniques include a number of complex engineering technologies that allow us to better understand the pathophysiology of diseases treated with DBS, and the resultant effects of stimulation on the system as a whole. In particular, much work has been done looking at whole-brain network activity resulting from electrical stimulation. As previously mentioned, it is unclear as to how high-frequency DBS exerts its effects on surrounding structures. Thus, mathematical algorithms and computational models have been devised to help answer this question.

In 2004, Rubin and Terman (2004) created a computational network model to demonstrate how DBS of the STN results in the disruption of downstream pathological thalamic rhythms in PD. They did this by first creating mathematical equations and algorithms representative of the individual cell properties of each neuronal population with respect to the channels and molecular transporters comprising the cell membranes, the membrane capacitance, current flux across membranes, and additional factors in order to accurately model each cell type and its surrounding environment. They could then predict the various cell firing patterns under different circumstances depending on the variables changed. In this way, the authors evaluated the neuronal firing patterns of the STN, GP externa, GPi, and thalamus during simulation of three states: (1) normal conditions, in which GPi output to the thalamus is irregular with minimal effects on thalamic cells; (2) parkinsonian conditions, during which GPi cells, with output to the thalamus, fire in bursts at a tremor frequency, resulting in disruption of the processing capabilities of the thalamus; and (3) conditions of STN DBS in PD, causing subthalamopallidal projections that result in high-frequency tonic firing of the GPi, as opposed to the low-frequency bursting, which ultimately allows the thalamus to resume normal activity. From this model, the authors determined that STN DBS resulted in normalization of aberrant basal ganglia oscillations and thus abnormal basal ganglia output to the thalamus, ultimately restoring physiologic thalamic relay capabilities (Hahn and McIntyre, 2010).

Additional models have since been developed to further elucidate the role that STN DBS has on GPi bursting and subsequent thalamic processing. In 2010, Hahn and McIntyre (2010) looked at a model similar to that of Rubin and Terman, however, they attempted to parameterize a subthalamopallidal network model incorporating presumed cortical and striatal inputs thought to influence abnormal beta rhythms within the basal ganglia. They did this by training the model to fit *in vivo* microelectrode recordings from the basal ganglia of parkinsonian non-human primates (Hahn and McIntyre, 2010). As a result, the authors sought to more accurately predict the dynamic, multi-step cortico-striatal-thalamic network in a DBS model for PD. This complex analysis demonstrated that normalization of aberrant GPi bursting depends on the volume of STN tissue activated and may require a threshold reduction of bursting for therapeutic effects. It also reinforced the principle that extensive signaling pathways and networks, rather than isolated cell-to-cell interactions, play a large role in the etiology of disease states, as evidenced by the cortical and striatal inputs to the basal ganglia, which were reflected by the model. Therefore, using computational modeling systems, our understanding of the pathophysiology of diseases can be improved, which may in turn, allow us to more effectively treat diseases.

One further example of how computational modeling can be used to represent and predict multi-faceted systems within the brain is seen in a study by Humphries and Gurney (2012). They also utilized intricate models to assess the effects of STN DBS on the basal ganglia in PD (Humphries and Gurney, 2012). The authors hypothesized that STN DBS resulted in both increased and decreased firing rates within the basal ganglia output nuclei, contributing to the therapeutic effects of treatment. In their model, they replicated the diversification of responses from a primate STN DBS study, and showed that the mixed basal ganglia output responses underwent a step-wise change with therapeutic DBS frequencies (>100 Hz), which did not occur in optogenetic models of direct STN stimulation. They concluded that the efficacy of DBS was due to mixed effects of stimulation resulting in a combination of both stimulatory and inhibitory output from the basal ganglia in response to electrical STN stimulation unique to DBS at therapeutic thresholds. Once again, this highlights the complexity of processes concomitantly occurring as a result of DBS, leading to widespread network effects.

#### Volume of Tissue Activated

**Figure 2:**
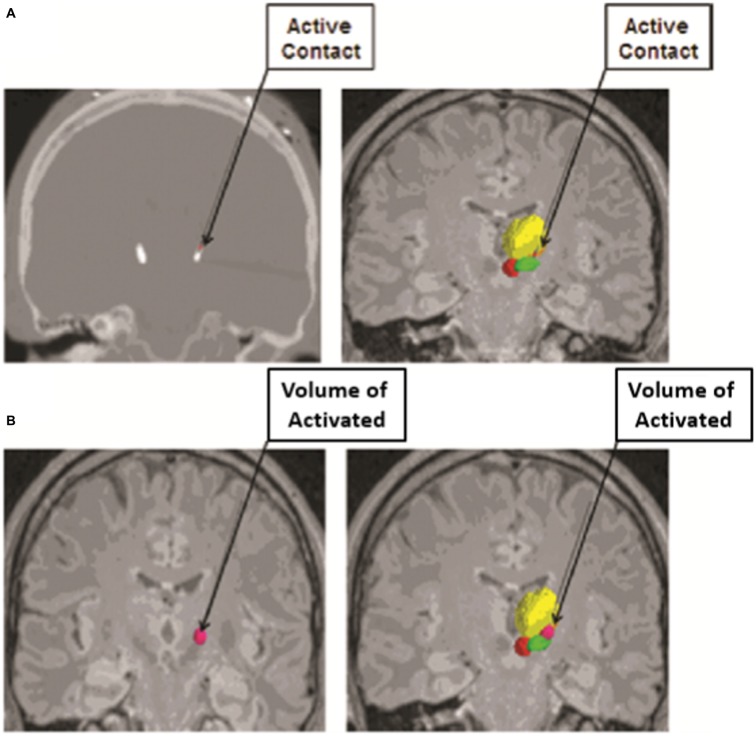
**Volume of Tissue Activated as determined from an anatomical model based on imaging data from subjects who have undergone deep brain stimulation (DBS) coupled with finite element modeling (FEM) to calculate the spread of electrical current through the tissue from the active electrode contact. (A)** The active contact of each electrode was determined from postoperative programming sessions, and was defined as the cathodal electrode for either monopolar or bipolar stimulation. The active contact is shown on a postoperative computed tomography (CT) scan (left) and on image fusion on magnetic resonance imaging (MRI; right). **(B)** The volume of tissue activated was calculated from each patient’s specific stimulation parameter settings by using a custom Python-based program from Python libraries. The volume of tissue activated is shown in dark pink alone (left) and in conjunction with the contored nuclei (right). The relevant nuclei were contored and include the thalami *(yellow)*, the red nuclei *(red)*, and the STN *(green)*; the active contact and the volume of tissue activated were also contored *(pink)*. Adapted from Sweet et al. (2014a).

Another computational modeling technique that has played a critical role in our understanding of the effects of DBS is the concept of visualizing the extent of tissue that is influenced by DBS electrode stimulation, or the volume of tissue activated (Figure 2; Sweet et al., 2014b). In 2006, Miocinovic et al. (2006) developed a computational model in parkinsonian macaques of STN DBS. This model predicted the pattern of axonal activation during electrode stimulation of the STN, and demonstrated that while both STN and GPi fibers were activated, only activation of the STN neurons correlated with clinically therapeutic stimulation (Miocinovic et al., 2006).

The method by which such computational modeling systems determine the volume of tissue activated involves finite element modeling (FEM). FEM combines an anatomical model based on imaging data from subjects who have undergone DBS with an electrical model to calculate the electrical field from the DBS electrode. The FEM data can then be used to calculate the voltage spread of the electrical stimulation. This depends on the location of the electrode and the composition of the surrounding tissue, which is in turn evaluated by the electrode capacitance and impedance, as well as by the stimulation parameters used with programming of the electrode. Ultimately, the voltage distribution within the tissue is established from the FEM and the total amplitude of current is divided between the contacts of the electrode (Butson and McIntyre, 2005, 2008; Miocinovic et al., 2006). Axonal activation can then be predicted by again using an anatomical model to distribute axons in a grid around the DBS electrode and calculating the voltage spread produced by each active contact to find the threshold for generating an action potential within each surrounding axon. The volume of tissue activated is then predicted from the relationship between the electrical field and the evoked action potentials in the axon models (Butson and McIntyre, 2008).

Such modeling algorithms are not only able to accurately predict the volume of tissue activated, but can also determine its effect on clinical outcome. Maks et al. (2009) evaluated 10 patients with PD who underwent unilateral STN DBS using conventional preoperative imaging and intraoperative neurophysiology. The results were then superimposed to a brain atlas and fused with postoperative imaging of the electrodes and the volume of tissue activated calculated with a diffusion tensor stimulation model, followed by assessment of clinical outcomes. The authors found that the therapeutic benefits of stimulation were greatest with the active contacts near the dorsal border of the STN. Similarly, in 2011, Mikos et al. (2011) used computational modeling to analyze of the association between the volume of tissue activated and verbal fluency in PD patients who underwent STN DBS, finding a subtle decline in verbal fluency with stimulation of non-motor regions of the STN. Thus the volume of tissue activated can be assessed relative to the surrounding anatomy in the brain to better determine which structures are actively being stimulated in an effort to improve patient outcomes.

#### Limitations of Computational Modeling

It should be noted that there are limitations to computational modeling techniques. With respect to the modeling of network activity, numerous simplifications and assumptions are made and are therefore limitations of the model. For instance, the input and output connections to and from the basal ganglia likely ignore many fiber tracts in order to focus on the pathways under investigation. As a result, networks that are potentially involved in sensorimotor signals to the thalamus may also be neglected (Rubin and Terman, 2004). In addition, field effects and variations due to electrode positioning are also ignored, and each cell is treated as a single compartment, again simplifying the model substantially (Rubin and Terman, 2004; Hahn and McIntyre, 2010). Many of these models also look at an open-loop system, studying the effects of a unidirectional pathway or network. However, in actuality, there are likely closed-loop, feedback interactions that are not represented with such models (Hahn and McIntyre, 2010).

Similarly, calculations for the determination of the volume of tissue activated are also based on several assumptions and simplifications regarding the properties of the activated neurons. Preliminary studies to calculate the volume of tissue activated were initially based on the properties of rat STN neurons. Attempts were then made to approximate *in vivo* conditions of Parkinson macaques. In addition, activated neuronal populations were presumed to be uniform in their membrane dynamics, myelination, morphology, and projection pattern. Yet we know such homogeneity does not exist *in vivo*. As a result, the true thresholds for producing action potentials and thus the overall volume of tissue activated could vary somewhat compared to the predicted model. Finally, studies investigating the volume of tissue activated looked at the white matter tracts surrounding the STN that were felt to be the most likely contributing tracts to the therapeutic effects of STN DBS, while many other nearby tracts were ignored. Also ignored were the potential effects of antridromic stimulation of afferent fibers projecting to the STN and the effects of adjacent glial cells that could influence the extracellular environment and thus the effects of current spread and axonal activation (Miocinovic et al., 2006).

### Additional Engineering Developments

#### Novel Stimulation Parameters

Many of the computational models predicting network activity with DBS have looked at the effects of high-frequency stimulation. In fact, as demonstrated by Humphries and Gurney (2012), the therapeutic effects of DBS are thought to occur with continuous stimulation at frequencies greater than 100 Hz. However, altering the parameters of the stimulation delivered could have significant results. Computational analyses using predictive models of stimulation have demonstrated that the volume of tissue activated is determined by both the stimulation parameters and the physical properties of the electrodes (Butson and Mcintyre, 2006). This has substantial implications regarding the importance of modulating stimulation settings to influence patient outcomes. Studies exploring innovative stimulation parameters such as high-frequency or patterned stimulation may have additional benefits compared to conventional stimulation. Moreover, such novel patterns of stimulation may be more effective in disrupting abnormal beta band oscillations within the cortico-basal ganglia network that are thought to be involved in the symptomatology of PD (Hurtado et al., 1999; Meijer et al., 2011; Grant and Lowery, 2013; Kang and Lowery, 2013; McIntyre and Foutz, 2013).

### Closed-Loop DBS and Current Steering

The delivery of stimulation can also be affected by the design of implanted DBS electrodes. New types of electrodes with recording capabilities have been studied such that changes in the environment surrounding the electrode can be assessed. Such changes include the recording of local field potentials, which could be used to correlate beta band oscillations with activity and posture in PD patients (Quinn et al., 2015) or even the characteristic chemical composition of the environment (Bennet et al., 2016). Recording electrodes such as these would then transmit the information to a pulse generator capable of analyzing the data, which would in turn deliver stimulation in response to the recorded information (Quinn et al., 2015; Bennet et al., 2016). This closed-loop responsive form of stimulation, may ultimately be used to further impact patient outcomes.

**Figure 3:**
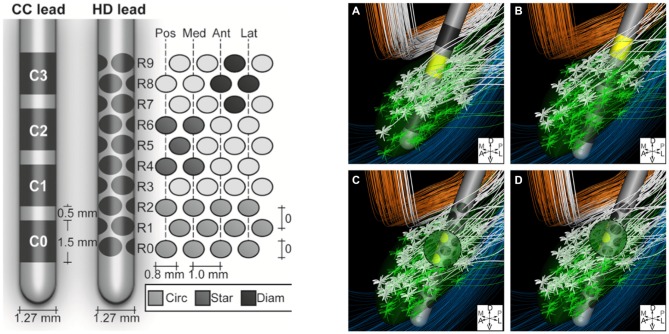
**Left: Conventional cylindrical four contact lead (CC) compared to high density lead design (HD).** Right: An example of diffuse fiber activation with conventional DBS **(A,B)** vs. selective activation of fiber tracts with current steering **(C,D)**. Reprinted with permissions from Van Dijk et al. (2015).

The volume of tissue activated can also be manipulated with changes in electrode design. For instance, as discussed by Butson and McIntyre (2008), shaping the volume of tissue activated by selectively activating specific fiber tracts via “current-steering” may better alleviate symptoms experienced by patients due to their disease. For example, in situations in which traditional STN DBS produces both therapeutic effects as well as concurrent side effects due to the undesired activation of adjacent fiber tracts like the internal capsule, current steering could be applied. This would enable directing the spread of current to avoid the activation of certain nearby structures, while activating the intended target. Currently utilized DBS electrodes have four cylindrical contacts, yet new electrode designs with more contacts of different shapes, capable of delivering stimulation in specific and variable configurations, are being developed (Figure 3; Van Dijk et al., 2015). These designs take advantage of the innumerable possible electrode orientations to maximize the efficacy of stimulation with surrounding anatomical structures (Figure 3; Beriault et al., 2012; Van Dijk et al., 2015). This notion of current-steering has given rise to multiple investigations (Contarino et al., 2014; Bour et al., 2015; Timmerman et al., 2015; Willsie and Dorval, 2015) and combining these engineering designs with computational models, such as those looking at the volume of tissue activated, could also prove to be useful in surgical planning and subsequent electrode programming. However, additional studies with long-term follow-up will be necessary to better determine the efficacy of such technologies.

### Advanced Neuroimaging Technologies

#### Diffusion-Weighted Imaging and Tractography

The development of sophisticated imaging techniques has also enhanced our understanding of the complex circuitry of neural networks in the brain and the role of DBS in influencing such networks. As previously discussed, many of the effects of DBS are likely due to the involvement of white matter tracts comprising interconnected functional systems traveling between various intracranial structures. Neuroimaging modalities measuring metabolism and blood flow have demonstrated diffuse patterns of seemingly distant regions of the brain being simultaneously activated with DBS, thus supporting this notion of stimulation affecting widespread connectivity (Mayberg, 1997; Grafton et al., 2006; Laxton et al., 2010; Kahan et al., 2012; Figee et al., 2013). However, these studies do not directly show the actual white matter pathways thought to be involved.

**Figure 4:**
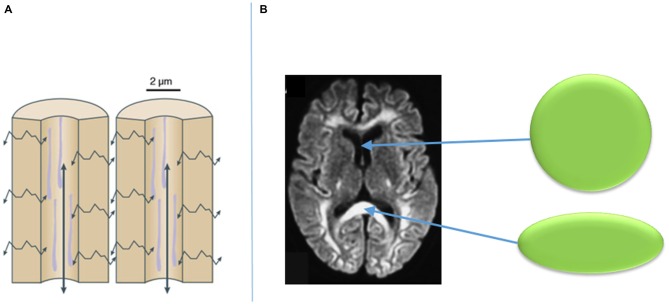
**Diffusion of water along cellular barriers in brain rather than across them (A), and examples of tensors with isotropic diffusion in CSF (represented by sphere) and anisotropic diffusion in callosal fibers (B).** Adapted from Klein (2013).

Visualization of such fibers is now in part feasible with the use of diffusion-weighted imaging (DWI) MRI sequencing and tractography. DWI is a mode of MRI that illustrates the spontaneous diffusion of water within the brain. Since water diffuses easily along cell barriers rather than across them, one can assume that the diffusion of water throughout the brain represents the anatomical course of neuronal axons. Thus, by assessing water diffusivity in living tissue, the approximate location of axonal pathways can be estimated, and the likelihood of their connections to various structures can be established (Henderson, 2012; Klein, 2013; Auriat et al., 2015). From DWI, a diffusion tensor can be established within a given voxel on MRI. A diffusion tensor can take a perfectly spherical shape when there is no water diffusion, such as the cerebrospinal fluid within a ventricle (Figure 4; Klein, 2013). However, the tensor can also become cylindrically-shaped when the water is diffusing in a given direction, such as along an axon as part of a group of white matter fibers (Figure 4; Klein, 2013). The more directions that are added to the DWI sequencing, the more specific the directionality of the diffusion tensor of a given voxel becomes. From the diffusion tensors, which are derived from DWI sequences, comes diffusion tensor imaging (DTI), which provides a voxel-to-voxel based estimation of the three-dimensional orientation of an axonal fiber (Auriat et al., 2015).

However, DTI has numerous limitations. Firstly, each diffusion tensor is only an estimation of the approximate directionality of water within a given voxel. As such, each tensor is only representative of a small portion of a single axonal fiber, and thus is not a representation of the entire axon or a group of axons comprising a fiber tract. Therefore, DTI may not even depict the actual anatomical course of a neuronal axon. As a result, any interpretation of the course of an axon or group of axons from DTI is relatively subjective (Auriat et al., 2015). Furthermore, DTI is limited by its technical acquisition, including poor resolution of images, poor signal-to-noise ratio, involuntary movements from subjects, and other related variables (Henderson, 2012).

A natural extension of DTI that attempts to correct for its limitations is fiber tractography. While DTI approximates the main nerve fiber direction within a given voxel, tractography attempts to assess all the voxels between two or more regions of interest in an effort to discern the entire course of axons comprising a white matter fiber tract (Klein, 2013). In order to achieve this, tractography uses the DWI data and a series of algorithms to produce a proposed fiber tract. By defining starting and ending seeds, or regions of interest, post-imaging analysis can be performed using various computer software platforms to determine the predominant fiber pathways coursing between the assigned seeds. However, it should be noted that tractography results are highly dependent upon the algorithms used, and larger white matter tracts are easier to visualize than smaller, unknown tracts (Coenen et al., 2012; Klein, 2013). In addition, sharply decussating tracts may be more difficult to identify than tracts without sudden or significant changes in direction (Coenen et al., 2012).

**Figure 5:**
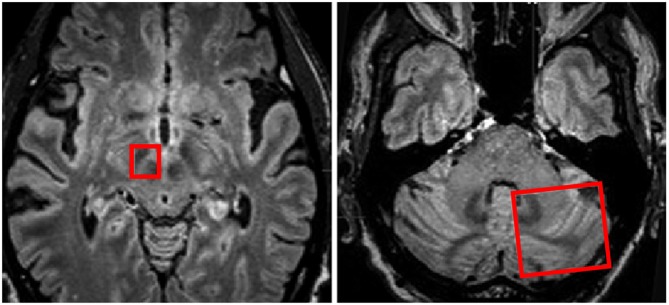
**Regions of interest (red boxes) shown on MRI in axial planes to include the right subthalamic nucleus (STN) and Red Nucleus and the contralateral cerebellar hemisphere for investigation of white matter tracts traversing between the two regions of interest.** Adapted from Sweet et al. (2014a).

**Figure 6:**
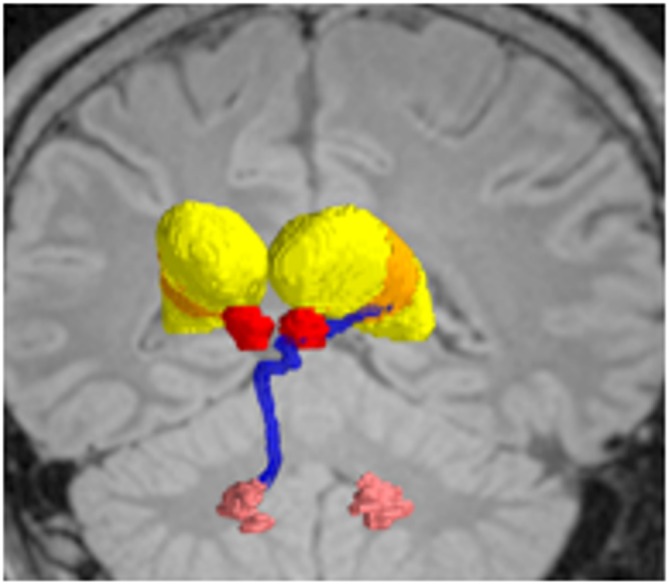
**Deterministic fiber tractography, demonstrating the dentatothalamaic tract (DTT; blue) in patients with tremor-predominant Parkinson’s disease (PD).** The relevant nuclei were contored and include the thalami *(yellow)*, the red nuclei *(red)*, and the dentate nuclei *(peach)*. Data from from Sweet et al. (2014a).

Thus tractography provides an anatomical three-dimensional model of white matter fiber tracts. There are two types of tractography, deterministic and probabilistic. Deterministic tractography is the more straightforward of the two and involves a streamline algorithm based on thresholds for collinearity of primary diffusion direction among adjacent voxels to establish a putative axonal pathway. These algorithms, such as FACT (fiber assignment by continuous tracking) create continuous tubular structures that are representative of white matter tracts between two or more regions of interest (Figures 5, 6; Sweet et al., 2014b). However, this is an all-or-none calculation, and not all of the tracts identified using this technique will in fact exist, which again represents a substantial limitation. There is also, as noted above, particular ambiguity in areas with less anisotropy such as decussating fibers (Coenen et al., 2012; Klein, 2013). Thus, deterministic tractography works well for highly anisotropic tensors, but may be quite inaccurate for areas with less anisotropy. While any potential error in estimation may be evident for well-established white matter tracts, such as the corticospinal tract, the degree of inaccuracy is indeterminable for tracts that are not well established. Therefore, adding to the above limitations of deterministic tractograpy, is the major disadvantage that the error or confidence in the estimation of a tracts is unknown (Coenen et al., 2014).

In contrast, probabilistic tractography attempts to overcome the limitations of deterministic tractography by explicitly characterizing the confidence with which connections may be established through the diffusion data itself (Coenen et al., 2012). Thus, probabilistic tractography uses advanced mathematical algorithms to produce an output of connection probability values to determine the statistical probability of a fiber tract running through a given set of voxels or between two anatomic structures based on the diffusion model (Coenen et al., 2012; Klein, 2013). This often requires the use of computer software programs such as FSL, which calculate and produce a color scale to indicate the probability of a given streamline connecting a start point to other voxels within the brain (Coenen et al., 2014). The same predictability could only be done with deterministic tractography by performing the fiber tracking on multiple patients and combining the results to determine a probability (Coenen et al., 2014). Once again, the primary limitation of tractography is its intrinsic nature of estimating the course and location of a fiber tract from the diffusion imaging data. However, whereas deterministic tractography cannot assess the degree of estimation error, probabilistic tractography is able to more precisely predict the likelihood of accuracy of a given fiber tract.

From a DBS standpoint, tractography makes it possible not only to visualize white matter pathways in the brain, but also to postulate how modulation of these pathways might lead to improved treatment outcomes. For example, both of the common DBS targets for PD (STN and GPi) are gray matter structures, and stimulation of either target results in notable improvement in motor symptoms (Walter and Vitek, 2004; Follett et al., 2010). Tractography has made it possible to view the axonal pathways surrounding each structure that may also be activated, such as subthalamopallidal and pallidothalamic pathways, as well as other connections to regions of the cortex, thalamus, and brainstem (Vanegas-Arroyave et al., 2016) and this activation may be responsible to some degree for the therapeutic effects of DBS. Similarly, studies assessing white matter tracts associated with the VIM thalamus, such as the dentantothalamic tract, indicate changes in tremor patients compared to controls that may account for some of the efficacy of DBS of this target (Klein et al., 2011). By identifying specific white matter pathways like the dentothalamic tract, which is thought to play a role in the pathogenesis of tremor, it is possible to determine whether there is any benefit to tract-specific targeting using DBS (Kwon et al., 2011; Klein et al., 2012; Coenen et al., 2014; Sweet et al., 2014b; Schlaier et al., 2015). In the same way, a better understanding of the involvement of white matter tracts in the pathophysiology of disease using tractography techniques can help direct future therapies. Using tractography to examine fiber networks in the brain of patients with psychiatric or cognitive disorders may allow identification of new targets in the brain for the treatment of various disorders. For example, Gutman et al. (2009) used DTI and probabilistic tractography to investigate two DBS white matter targets for the treatment of depression, the subcallosal cingulum bundle and the anterior limb of the internal capsule, and concluded that these targets are involved in distinct neural networks that overlap, explaining the benefit of DBS of each target in patients with depression. Likewise, Fernandes et al. (2015) used advanced tractography combined with whole-brain anatomical parcellation to establish connectivity “fingerprints” of successful and unsuccessful DBS targets for chronic pain, a technique which offers promise for discovery of new neuromodulation targets for other neuropsychological disorders. Boccard et al. (2016) used probabilistic tractography to demonstrate that connectivity with medial forebrain bundle and precuneus were associated with good and bad outcome, respectively, among patients treated with DBS of the anterior cingulate for chronic pain. The targeting of temporal fiber pathways for disorders of memory and cognition is also actively under investigation (Hamani et al., 2007; Laxton et al., 2010; Suthana et al., 2012; Miller et al., 2015). Incorporating tractography imaging techniques to better visualize neural pathways may prove to be invaluable in such targeting endeavors.

#### Resting-State Functional Connectivity MRI and Connectonomics

It is also worth discussing additional imaging technologies that have been developed to further assess connectivity, such as resting-state functional-connectivity MRI (rs-fcMRI). This technique allows for the visualization of neural networks within the brain by assessing fluctuations in blood oxygenation (Fox et al., 2014). Spontaneous alterations in the BOLD has been correlated with known functional networks within the brain, and may demonstrate continuous activity between various intracranial regions, thus representing connectivity (Faria et al., 2012). Such networks have been shown to be active in the absence of sensorimotor or cognitive tasks in fMRI studies, and deactivate during such goal-directed tasks (Ongur et al., 2010). As a result, these pathways have been termed the default-mode network (DMN) and are implicated in ongoing, default functions of the brain (Ongur et al., 2010). Thus, rs-fcMRI allows us to assess the DMN in patients as a surrogate for connectivity. Moreover, rs-fcMRI also enables visualization of brain structures more easily than tractography, which is derived from DWI MRI sequences rather than structural MRI sequencing. This also allows for structural connectivity-based parcellation to be incorporated into the imaging analysis. The parcellated functional connectivity approach is a natural extension of rs-fcMRI due to the spatial dimensionality visible in rs-fcMRI. This entails identifying brain structures and clustering voxels around such structures (Faria et al., 2012). This results in a more clearly depicted relationship between functional connectivity and structural anatomy. Such analysis can be taken a step further to include multi-modal analysis, combining DWI-based connectivity with rs-fcMRI connectivity using a parcel-to-parcel approach (Faria et al., 2012).

Data from rs-fcMRI pertaining to the DMN can be used to help draw conclusions regarding the wiring diagram of the brain, or the connectome. By modeling the DMN derived from BOLD signals on rs-fcMRI, lesions or disruptions of connections can be replicated to determine the resultant impact such changes would produce on the DMN (Hart et al., 2016). Thus by weakening specific projection fibers, as one might see in injuries induced by traumatic brain injury, one can predict the resultant effects this wound have on the connectome as a whole (Hart et al., 2016). Similarly, by strengthening certain connections, we can speculate as to the impact that DBS might have on the connectome or DMN.

#### Limitations of Connectivity Analyses

However, like any novel modality, limitations in both tractography and rs-fcMRI do exist. With respect to tractography, as previously discussed, the visualization of fiber tracts is based on numerous assumptions regarding the diffusion properties of water within the brain. Firstly, the premise of tractography relies on the presumption that the spontaneous flow of water in the brain only occurs along the course of an axon. Moreover, the tensors are themselves estimations of the mean vector of water diffusivity within a given voxel. Lastly, the mathematical algorithms that generate proposed fiber tracts are at best educated estimations of the presence of a given tract.

In addition, limitations of rs-fcMRI should also be noted. While there is a demonstrable correlation between rs-fcMRI and white matter connections, rs-fcMRI is still a surrogate for connectivity based on a suspected DMN. In addition, the exact causal relationship between two or more regions with increased BOLD can only be postulated, and polysynaptic interactions may play a larger role then suspected. This could thus be a misrepresentation of a particular connection or relationship in the brain, which could be misleading if used for target guidance in DBS (Fox et al., 2014).

### Integration of Novel Technologies and Future Applications in Target Selection

**Figure 7:**
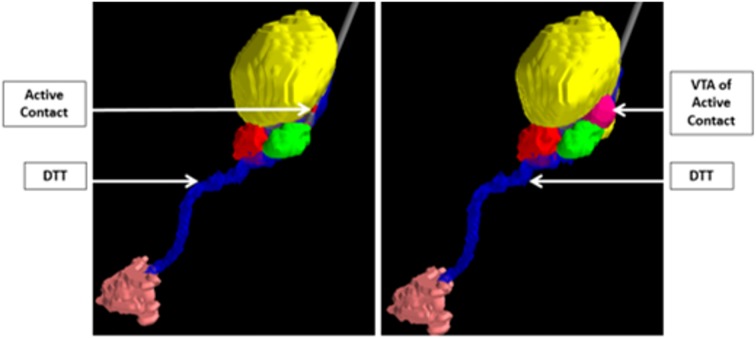
**Integration of computer modeling techniques and tractography to demonstrate that the volume of tissue activated of the active STN electrode contact includes the DTT in patients with tremor-predominant PD.** The relevant nuclei were contored and include the globus pallidus and putamen *(blue)*, the thalami *(yellow)*, the red nuclei *(red)*, the STN *(green)*, and the dentate nuclei *(peach)*. Data from Sweet et al. (2014a).

Ultimately, the integration of computational modeling techniques and novel imaging technologies can perhaps be used to validate hypotheses regarding mechanisms of disease states and implicated networks, and thus aid in the discovery of new surgical targets. For example, in a study by Sweet et al. (2014b), patients with advanced PD who underwent STN DBS were assessed using deterministic tractography from preoperative imaging to identify fiber pathways extending between the cerebellum and the basal ganglia believed to be associated with the pathophysiology of the tremor component of PD. The findings from the tractography were then combined with postoperative computational modeling to determine the volume of tissue activated based on the therapeutic electrode contacts. The study showed that in patients with tremor-predominant PD, the volume of tissue activated tended to involve the dentatothalamic tract (Figure 7; Sweet et al., 2014b), supporting the idea that this tract contributes to tremor in PD. The findings also suggest that specific targeting of the dentatothalamic tract may better alleviate symptoms in tremor-predominant patients, and may thus represent a form of patient-specific DBS targeting.

Other studies have similarly integrated computational modeling, tractography, and patient outcomes. Specifically, the dentatothalamic tract has been assessed in PD and ET patients by several groups, using both deterministic and probabilistic tractography (Klein et al., 2012; Coenen et al., 2014; Sweet et al., 2014b; Schlaier et al., 2015), and combined with computational models to further validate this concept of patients-specific targeting to treat tremor symptomatology. Outcome data from patient-specific targeting techniques can also be combined with computational models to construct a probabilistic stimulation atlas that incorporates advanced computational modeling with clinical outcomes to determine the ideal target based on a patient’s particular symptoms (Butson et al., 2011). Though further studies are needed, tractography and predictive modeling modalities may be able to be used to rather directly target specific pathways in a patient-specific manner .

Finally, the combination of computational models of electric field patterns with tractography could help us to better predict which specific white matter tracts are being activated for the treatment of psychiatric, cognitive, and neurologically-mediated disorders. Lujan et al. (2013), combined probabilistic tractography and electrical field modeling to help predict connectivity in patients with depression. The authors looked at white matter tracts within the subcallosal cingulum bundle who underwent DBS to look at which electrodes would be the most useful in treatment of the depression based on the pattern of current delivery and activation of the associated fibers using computational modeling. Similarly, Coenen et al. (2012) demonstrated the involvement of the medial forebrain bundle in depression using tractography, and utilized computational modeling to show how a new DBS target stimulating the medial forebrain bundle would potentially activate more white matter tracts associated with the pathophysiology of the disease than currently investigated targets. As more disease entities are studied for potential treatment using DBS, such as other psychiatric disorders, memory and cognitive dysfunction, and epilepsy (Fisher et al., 2010; Sankar et al., 2014; Miller et al., 2015), the applications for these techniques will undoubtedly grow as well.

## Conclusion

Improvements in surgical targeting, computational modeling, engineering, and neuroimaging techniques have greatly enhanced our understanding of the pathophysiology of various diseases, allowing for the effective treatment of such conditions using DBS. In the future, it is likely that these novel technologies will have further applications in DBS surgery, including patient-specific targeting and the discovery of new targets for the treatment of neurological, psychiatric, and even cognitive disorders.

## Author Contributions

JPM, JAS, JP, and FG wrote and edited the article.

## Conflict of Interest Statement

The authors declare that the research was conducted in the absence of any commercial or financial relationships that could be construed as a potential conflict of interest.
